# Predicting Risk of Lymph Node Metastasis in Neuroendocrine Carcinoma of Cervix: A Validated Nomogram Incorporating Neuroendocrine Markers and Clinical Parameters

**DOI:** 10.1002/cam4.71686

**Published:** 2026-03-06

**Authors:** Mingzhu Jia, Siyuan Zeng, Juan Zou, Huiling Chen, Changsheng Lin, Shuqi Yang, Jiangchuan Pi, Xue Xiao

**Affiliations:** ^1^ Department of Gynecology and Obstetrics West China Second University Hospital, Sichuan University Chengdu China; ^2^ Key Laboratory of Birth Defects and Related Diseases of Women and Children (Sichuan University), Ministry of Education West China Second University Hospital, Sichuan University Chengdu China; ^3^ Department of urology Chengdu Second People’s Hospital Chengdu China

**Keywords:** lymph node metastasis, neuroendocrine carcinoma of cervix, neuroendocrine markers, nomogram model

## Abstract

**Objective:**

Lymph node metastasis (LNM) is an important factor leading to poor prognosis of tumors. This study aims to predict the risk probability of LNM in neuroendocrine carcinoma of cervix (NECC).

**Methods:**

202 and 92 patients were included as the training cohort and the validation cohort respectively. Logistics regression analysis was conducted to determine the risk factors related to LNM in the training cohort. The validity of the model was evaluated by the calibration curve and the consistency index. The receiver operating characteristic curve was used to determine the optimal threshold for predicting the risk of LNM. Then, it compared the predictive ability of the different models and their ability to identify low‐risk patients.

**Results:**

Multivariate logistic regression analysis confirmed that the depth of stromal invasion (*p* = 0.029), parametrium invasion (*p* = 0.046), lymphovascular space invasion (*p* = 0.011), cervical‐uterine junction invasion (*p* = 0.046), and positive CD56 (*p* = 0.008) were the independent risk factors for LNM, which were included in the construction of the nomogram model. Both the internal and external calibration curves showed that the model fits well. The C‐index of the training cohort and the validation cohort in this developed model (0.894 and 0.92, respectively) was superior to other models. The optimal threshold of risk probability of LNM predicted by the model was 0.20. Based on this threshold, this model showed a good recognition ability to identify low‐risk patients.

**Conclusion:**

The nomogram model constructed by combining clinical parameters with neuroendocrine markers could effectively predict the risk probability of LNM in NECC and identify the low‐risk population.

## Graphical abstract

This study creates a nomogram model to predict risk probability of lymph node metastasis in NECC based on neuroendocrine biomarkers (CD56) and clinical factors. This model is superior to other existing models through the comparison. And it enables clinicians to identify low‐risk patients (risk probability threshold: 0.20) to guide the individualized range of operation, potentially sparing them unnecessary lymphadenectomy and its associated morbidity.

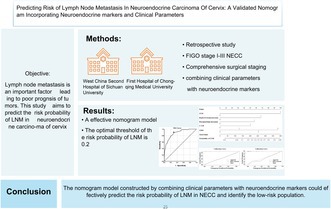

## 1 Introduction

Neuroendocrine carcinoma of cervix (NECC) is a rare and specific type of gynecological malignancy, and the incidence accounts for approximately 1%–5% of all cervical cancers [1, 2, 3]. Unlike other cervical squamous cell carcinomas at the same stage, NECC exhibits high aggressiveness and malignancy. It is highly susceptible to disease progression and tumor recurrence, particularly in high‐grade NECC. As a result, the overall survival rate of the patients is relatively poor. Even at the early stage of the disease, the prognosis remains unfavorable with a 5‐year survival rate of only 4%–51% [4, 5, 6].

Lymph node metastasis (LNM) is a primary factor resulting in the extremely poor survival rate in tumors. The risk of death increases by 3.5‐fold in patients with NECC [7]. The standard treatment for patients with early‐stage cervical cancer is synchronized chemo‐radiotherapy or radical hysterectomy with pelvic and/or para‐aortic lymph node dissection [8]. Adjuvant therapy is usually recommended when postoperative pathology reveals positive lymph nodes. Therefore, assessment of lymph node status is essential for determining the treatment strategy [9]. For early‐stage patients eligible for radical surgery, the guidelines have specified the indications for lymphadenectomy, including stage 1A1 with lymphovascular space invasion and stage IA2‐IIA. Lymphadenectomy has always been used as the standard method for evaluating lymph node status [10]. However, the therapeutic value of this procedure remains controversial [9]. Only 15%–25% of patients with early‐stage cervical cancer develop LNM. A large proportion of these patients undergo unnecessary lymph node dissection and suffer from surgery‐related complications, including lymph cysts, lymphedema, vascular injury, nerve injury, and infection [11]. Therefore, the ability to recognize patients with low probability LNM may be of significant value in the medical decision.

Unlike other types of cervical cancer, NECC exhibits a higher incidence of LNM compared to the common types of cervical squamous cell carcinoma [12, 13]. This pathologic heterogeneity further emphasizes the clinical necessity of developing a predictive model for LNM of NECC. NECC is characterized by over‐expression of neuroendocrine markers, including chromogranin A (CgA), neural cell adhesion molecule (CD56), neuron‐specific enolase (NSE), and synaptophysin (Syn). They are the most common neuroendocrine markers being detected by immunohistochemistry. These markers are often used to assist in the diagnosis and differential diagnosis of neuroendocrine carcinoma [3, 9, 14, 15, 16]. In recent studies, these neuroendocrine markers have been found to be significantly correlated with tumor recurrence and are identified as potential prognostic markers [17]. These findings suggest that neuroendocrine markers may serve as crucial candidate indicators of constructing a predictive model for LNM. In recent years, although a few studies have developed models to predict the risk of LNM in cervical cancer [18, 19, 20], these models lack specificity in assessing LNM of NECC. Moreover, most of the existing prediction models simply rely on traditional clinicopathological parameters. And it is very rare to construct a model to predict LNM of NECC by using traditional clinical parameters in combination with neuroendocrine markers.

Therefore, the aim of this study is to combine traditional clinical parameters and neuroendocrine markers to establish a novel model for predicting LNM of NECC. This model will quantify the probability of LNM in patients with NECC, thereby identifying patients who may benefit from lymphadenectomy and guiding the individualized lymph node dissection and the selection of neoadjuvant chemotherapy.

## 2 Materials and Methods

### 2.1 Research Population

The clinical data of patients with FIGO stage I‐III NECC hospitalized in the West China Second Hospital of Sichuan University and the First Hospital of Chongqing Medical University from January 1, 2010 to January 1, 2023 were retrospectively collected. Patients from the West China Second Hospital of Sichuan University were assigned to the training cohort and used to develop the nomogram. The patients from the First Hospital of Chongqing Medical University served as an external validation cohort. Baseline clinicopathological characteristics and clinical variables were compared between the two institutions to evaluate potential differences in patient characteristics. The inclusion criteria were as follows: (1) Patients who didn't receive preoperative radiotherapy or chemotherapy; (2) Patients who received surgical treatment; (3) Patients diagnosed as NECC by postoperative pathology. The exclusion criteria were as follows: (1) Patients who didn't receive standard surgical procedure; (2) Non‐primary tumor; (3) Patients with other malignant tumors; (4) Patients with fatal comorbidities; (5) Patients without complete data (including clinicopathological data and follow‐up information). The basic information, clinicopathological data and the expression levels of immunohistochemical marker were recorded. The study was approved by the Ethics Committees of West China Second Hospital of Sichuan University and the First Affiliated Hospital of Chongqing Medical University. The informed consent of all patients was in accordance with the ethical standards of the Declaration of Helsinki.

### 2.2 Treatment

All patients included in this study underwent a comprehensive surgical staging. It consisted of hysterectomy with bilateral salpingo‐oophorectomy plus systematic pelvic lymphadenectomy, with or without para‐aortic lymphadenectomy. Systematic pelvic and para‐aortic lymph node dissection was recommended for patients with high‐risk factors, such as lymphovascular space invasion (LVSI) and deep stromal invasion, and so on. According to relevant studies, removal of at least 10 pelvic lymph nodes with or without 5 para‐aortic lymph nodes was defined as effective lymph node dissection [21]. The decision of adjuvant therapy (complementary radiotherapy or even combined chemotherapy) is determined by international guidelines and multidisciplinary discussions after surgery [22].

### 2.3 Immunohistochemistry

Surgical specimens were immediately fixed in formalin after surgery and processed for pathological examination following standardized protocols. Results of tumor type, histologic grade, lesion size, extent of invasion and staining of immunohistochemical markers were initially assessed by a specialized junior pathologist and subsequently reviewed by a superior physician. Immunohistochemical results of CD56, Syn, P16, and CgA were independently evaluated by two experienced pathologists. Concordance was achieved if the percentage of positive tumor cells was consistent between both reviewers. If the initial estimates differed by more than 10% or +, the results were reevaluated to reach a consensus. Cases where consensus still could not be attained were adjudicated by a third pathologist until a consensus was reached. The immunohistochemical scoring criteria was semi‐quantitative. The intensity of immunohistochemical staining was exhibited according to a four‐point scoring system as follows: 0 (no positive staining), 1 + (weak or focal positive staining in < 5% of tumor cells), 2 + (moderate positive staining in 5%–50% tumor cells), 3 + (strong and diffuse staining in > 50% tumor cells), respectively.

### 2.4 Statistical Analysis

Statistical analyses in this study were performed using R software and IBM SPSS (version 26.0). Continuous variables were expressed as median or mean ± standard deviation, and the differences were compared using the student's *t*‐test or the rank‐sum test. Categorical variables were expressed as frequency and percentage, and the comparisons between groups were conducted by chi‐square test. With a two‐way test, *p* < 0.05 was statistically significant.

As NECC is a rare malignancy, sample size is inherently constrained. The candidate predictors were selected based on clinical relevance and prior evidence, and the number of variables included was restricted relative to the sample size. All potential predictor variables in the training cohort were initially assessed for their association with LNM of NECC using univariate logistic regression analysis. Then, the factors with *p* < 0.05 were further included in the multivariate logistic regression analysis, and the odds ratios for each factor were calculated to identify independent risk factors associated with LNM of NECC. Independent predictors identified in the multivariate logistic regression analysis (*p* < 0.05) were incorporated to establish a predictive nomogram model using R software. The predictive performance of the nomogram was evaluated through internal and external validation. The discriminative ability of the model was quantified by the consistency index (C‐index) and calibration curves. The C‐index can quantify the degree of consistency between the actual occurrence and the predicted probability of the event. The rate between 0.50 and 0.70 was a low level of accuracy, that between 0.71 and 0.90 was a moderate level of accuracy and above 0.90 was a high level of accuracy. Finally, the ROC curve was used to find the optimal threshold. The patients in the training cohort were categorized into high‐risk LNM group (High‐LNM group) and low‐risk LNM group (Low‐LNM group) based on the optimal threshold. The ability of different models to discriminate high‐risk and low‐risk patients was compared by calculating the proportion of low‐risk group, sensitivity, specificity, positive predictive value (PPV) and negative predictive value (NPV). Sensitivity was defined as the proportion of patients with LNM correctly identified by the model among all patients with LNM. Specificity was defined as the proportion of patients without LNM correctly identified by the model among patients without LNM. PPV was defined as the proportion of patients classified as high‐risk LNM by the model who were actually accompanied by LNM. NPV was defined as the proportion of patients classified as low‐risk LNM by the model who were actually not accompanied by LNM.

## 3 Results

### 3.1 Clinicopathologic Data of Patients

**FIGURE 1:**
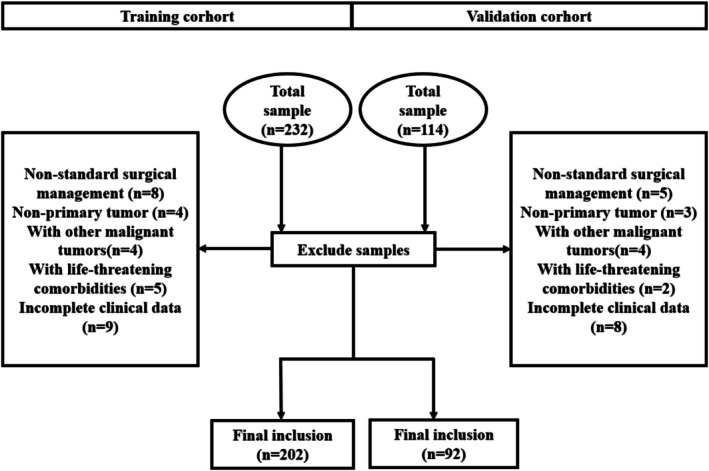
The screening process of patients in training cohort and validation cohort.

A total of 346 patients with NECC who were hospitalized and underwent surgery were collected. 294 patients were finally included in this study according to the inclusion and exclusion criteria. 202 patients from the West China Second Hospital of Sichuan University were divided into the training cohort, and 92 patients from the First Hospital of Chongqing Medical University were included as the validation cohort. The screening process was shown in Figure 1. The clinical and pathological characteristics of the training and validation groups were shown in Table 1. As shown in the results, there were no statistically significant differences in baseline characteristics between the two cohorts. This ensured that the external validation of the constructed model in the validation cohort would not be affected by confounding factors between the two cohorts. In the training cohort, 137 (67.8%) patients simultaneously underwent abdominal para‐aortic lymph node dissection, and LNM was observed in 58 (28.7%) patients, of which 8 had abdominal para‐aortic LNM. In the validation cohort, 56 (60.9%) patients concurrently underwent abdominal para‐aortic lymph node dissection, and LNM was observed in 26 (28.3%) patients, of which 3 had abdominal para‐aortic LNM. No cases of isolated para‐aortic LNM were observed in either cohort.

**TABLE 1:** Clinicopathological characteristics of the training and validation cohorts.

Variable	Training cohort	%	Validation cohort	%	*p*
	*N* = 202		*N* = 92		
Age (years)					0.877
Mean ± SD	45.86 ± 10.003		46.05 ± 10.422		
BMI (kg/m^2^)					0.776
Median (range)	22.30 (16.89–38.64)		22.63 (16.89–32.47)		
Pathological type					0.873
SCNECC‐alone	177	87.6	80	87.0	
SCNECC‐mix	25	12.4	12	13.0	
Histologic grade					0.940
HG‐grade	175	86.6	80	87.0	
LG‐grade	27	13.4	12	13.0	
Parametrium invasion					0.226
Yes	42	20.8	25	27.2	
No	160	79.2	67	72.8	
Nerve invasion					
Yes	188	93.1	83	90.2	0.398
No	14	6.9	9	9.8	
Depth of stromal invasion					0.503
< 1/2	73	36.1	37	40.2	
≥ 1/2	129	63.9	55	59.8	
C‐UJI					0.540
Yes	62	30.7	25	27.2	
No	140	69.3	67	72.8	
LVSI					0.450
Yes	147	72.8	63	68.5	
No	55	27.2	29	31.5	
P16					0.728
0	28	13.9	11	12.0	
1+	96	47.5	39	42.4	
2+	17	8.4	9	9.8	
3+	61	30.2	33	35.8	
Syn					0.227
0	25	12.4	10	10.9	
1+	95	47.0	41	44.6	
2+	39	19.3	12	13.0	
3+	43	21.3	29	31.5	
CgA					0.110
0	76	37.6	28	30.4	
1+	73	36.1	30	32.6	
2+	24	11.9	10	10.9	
3+	29	14.4	24	26.1	
CD56					0.217
0	89	44.1	36	37.0	
1+	47	23.3	26	29.3	
2+	44	21.8	14	15.2	
3+	22	10.8	16	18.5	
Scope of lymphadenectomy					0.244
Only pelvic LNs	65	32.2	36	39.1	
Pelvic + para‐aortic LNs	137	67.8	56	60.9	
Number of LNs removed					0.400
Median (range)	28 (10–82)		27 (10–60)		
LNM	58		26		0.937
Only pelvic LNM	50		23		
Pelvic + para‐aortic LNM	8		3		

Abbreviations: BMI, body mass index; C‐UJI, cervical‐uterine junction invasion; HG‐NECC, high grade‐neuroendocrinal carcinoma of cervix; LG‐NECC, low grade‐neuroendocrinal carcinoma of cervix; LNM, lymph node metastasis; LVSI, lymph vascular space invasion; SCNECC, small cell neuroendocrine carcinoma of the uterine cervix; SD, standard deviation.

### 3.2 Logistic Regression Analysis of Factors Predicting LNM in NECC

In this study, traditional clinical parameters and neuroendocrine markers that were relevant to LNM of NECC were screened by univariate and multivariate logistic regression analyses (Table 2). The results of univariate logistic regression analysis showed that age, depth of stromal invasion, nerve invasion, parametrium invasion, LVSI, cervical‐uterine junction invasion (C‐UJI), and CD56 were correlated with LNM. Factors with *p* < 0.05 were further included in multivariate logistic regression analysis. The results showed that depth of stromal invasion (*p* = 0.029), parametrium invasion (*p* = 0.046), LVSI (*p* = 0.011), C‐UJI (*p* = 0.046), and positive CD56 (*p* = 0.008) were confirmed as the independent risk factors of LNM. Therefore, these five factors were further utilized to construct a prediction model for LNM of NECC.

**TABLE 2:** Univariate and multivariate analyses of risk factors predicting lymph node metastasis of NECC in the training cohort.

Variables	Univariate analysis			Multivariate analysis		
	OR	95% CI	*p*	OR	95% CI	*p*
Age (< 50 vs. ≥ 50)	2.283	1.224–4.255	0.009	1.318	0.554–3.136	0.533
Depth of stromal invasion (< 1/2 vs. ≥ 1/2)	12.420	4.273–36.099	< 0.001	3.837	1.148–12.827	0.029
Nerve invasion (yes vs. no)	2.686	0.898–8.037	0.047	1.643	0.390–6.912	0.498
Parametrium invasion (yes vs. no)	8.667	4.076–18.429	< 0.001	3.073	1.018–9.279	0.046
LVSI (yes vs. no)	16.308	3.823–69.558	< 0.001	8.259	1.631–41.809	0.011
C‐UJI (yes vs. no)	7.427	3.608–13.935	< 0.001	2.758	1.020–7.459	0.046
CD56						
0	1.000		< 0.001	1.000		0.008
1+	5.737	2.244–14.672	< 0.001	3.037	1.020–9.045	0.046
2+	11.089	4.346–28.297	< 0.001	6.271	2.072–18.976	0.001
3+	8.437	2.781–25.600	< 0.001	5.726	1.510–21.721	0.010

Abbreviations: C‐UJI, cervical‐uterine junction invasion; LVSI, lymph vascular space invasion; OR, odds ratio.

### 3.3 Construction of the Nomogram Model and Verification of Its Performance

**FIGURE 2:**
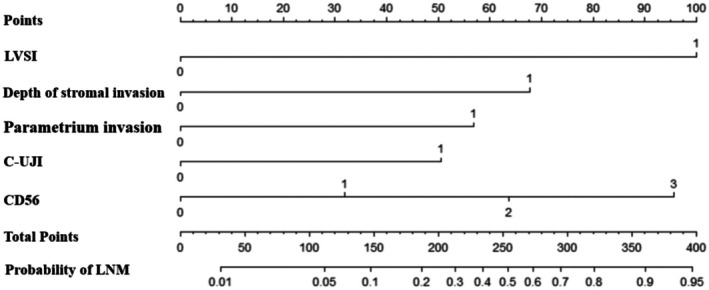
The nomogram model for predicting the lymph node metastasis of neuroendocrinal carcinoma of cervix after surgery (Each variable from the second row to the seventh row corresponds to a point value in the first row “point”. Then, sum the scores of each variable to obtain the “total point”, and finally derive probability of lymph node metastasis).

**FIGURE 3:**
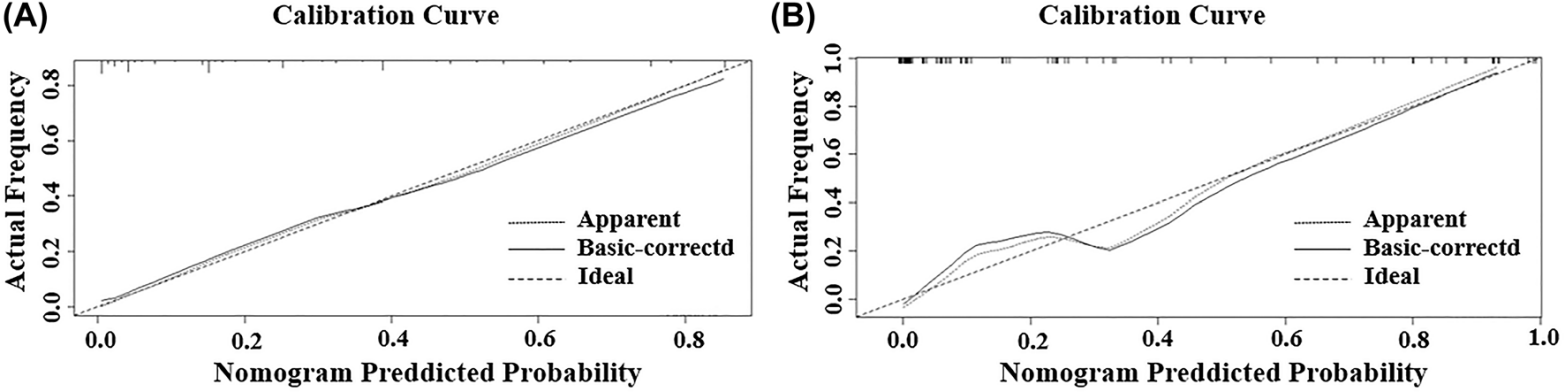
(A) The calibration curves of this nomogram model for lymph node metastasis of neuroendocrinal carcinoma of cervix in the training cohort. (B) The calibration curves of this nomogram model for predicting lymph node metastasis of neuroendocrinal carcinoma of cervix in the validation cohort.

The developed nomogram model for predicting the LNM of NECC was shown in Figure 2. It could estimate the risk probability of LNM of individual patients in a more accurate and simple way. In the nomogram, the relative contribution of each predictor for predicting LNM was represented by the length of its corresponding scale line. The specific score of its weight was quantified by the first line “points”, and then the score of each factor was summed to obtain a score meaning “total points”, and the “total points” corresponded to the patient's “probability of LNM”. As shown in the figure, the predictive value of LVSI and neuroendocrine marker was more significant compared with other classical clinicopathological parameters. This study used an independent external validation cohort from a different institution. The calibration curves of the model applied in both training and validation cohorts showed a good fit (Figure 3). This finding supported the robustness and generalizability of the model for predicting LNM in patients with NECC. Finally, the discriminative ability of several different models for LNM was compared using the C‐index. As shown in Table 3, the present model in this study had the highest C‐index in both the training cohort and validation cohort: the C‐index was 0.894 (95% CI, 0.823–0.925) and 0.920 (95% CI, 0.864–0.976), respectively. It suggested that the model integrating traditional clinical parameters and neuroendocrine marker exhibited superior discriminatory performance compared to other models that simply contained traditional clinical parameters.

**TABLE 3:** Discriminatory performance (C‐index) of different prediction models in the training and validation cohort.

Model	Author	Variable	Training cohort		Validation cohort		
			C‐index	95% CI	C‐index	95% CI	
Model A [20]	Yuan‐Run Deng et al.	Age, histologic subtype, tumor grade, tumor size, FIGO	0.723	0.707–0.738	External validation	0.747	0.690–0.804
					Internal validation	0.745	0.720–0.770
Model B [24]	Wei Liang Qian et al.	Age, tumor size, FIGO stage, ADC value, SCC level and the DL score	0.890	0.821–0.938		0.844	0.701–0.936
Model proposed in this study		Depth of stromal invasion, lymph vascular space invasion, cervical uterine junction invasion, parametrium invasion, CD56	0.894	0.823–0.925		0.920	0.864–0.976

### 3.4 The Optimal Threshold of the Risk Probability of LNM Predicted by This Present Model

**FIGURE 4:**
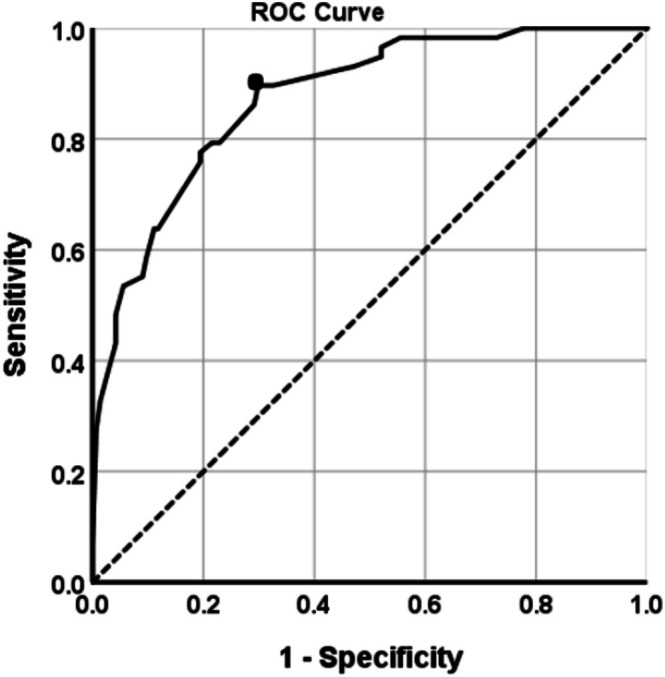
The ROC curve of the optimal threshold for probability of lymph node metastasis based on the developed nomogram model (Black dot: The area under the curve [AUC] at this point is the largest, which indicates the optimal threshold value of probability of lymph node metastasis predicted by the model is 0.20. Dotted line: Reference line. Solid line: The ROC curve of the established nomogram model).

With this proposed nomogram model, the risk probability of LNM could be calculated for each patient. Based on the ROC curve and Youden index, the optimal threshold of risk probability of LNM was determined to be 0.20 (sensitivity = 91.3%; specificity = 75.0%; area under the curve = 0.894; 95% CI [0.823–0.925]) (Figure 4). Based on this threshold, patients were divided into two groups: the Low‐LNM group (predicted risk probability ≤ 0.20) and the High‐LNM group (predicted risk probability > 0.20). According to the thresholds, 55.9% (113/202) (sensitivity = 91.3%, specificity = 75.0%, PPV = 59.6%, NPV = 95.6%) in the training group and 68.5% (63/92) (sensitivity = 88.5%, specificity = 90.9%, PPV = 79.3%, NPV = 95.2%) in the validation group were identified as the Low‐LNM group. In addition to the accuracy, the ability to identify the largest group of patients at low‐risk LNM was also critical for a model predicting the risk probability of LNM. The ability of different models to identify patients with low‐risk LNM was compared (Table 4). Through comprehensively comparing the indicators (percentage of patients with low‐risk LNM, sensitivity, specificity, PPV, and NPV), the model proposed in this study was significantly better than or close to the other models. It indicated that the ability of this model to identify patients who were with the low‐risk LNM was satisfactory.

**TABLE 4:** Discriminatory capacity of the constructed models in identifying patients with low‐risk LNM.

Model	Group	Proportion of low‐risk group	Number of LNM in low‐risk group	Sensitivity (%)	Specificity (%)	PPV (%)	NPV (%)
Model A [20]	Internal validation cohort	21.0% (389/1849)	12 (353 in total)	96.6	25.2	23.4	96.9
	External validation cohort	18.0% (82/455)	3 (64 in total)	95.3	20.2	16.4	96.3
Model B [24]	Training cohort	77.8% (98/126)	8 (30 in total)	73.3	93.8	78.6	91.8
	Validation cohort	74.4% (32/43)	3 (11 in total)	72.7	90.6	72.7	90.6
Model proposed in this study	Training cohort	55.9% (113/202)	5 (58 in total)	91.3	75.0	59.6	95.6
	Validation cohort	68.5% (63/92)	3 (26 in total)	88.5	90.9	79.3	95.2

Abbreviations: LNM, lymph node metastasis; NPV, negative predictive value; PPV, positive predictive value.

## 4 Discussion

The clinical management of NECC has always faced a challenge. Firstly, its rarity and biological heterogeneity result in a scarcity of high‐level evidence to guide practice. Secondly, it is difficult to accurately assess the risk of LNM by the existing tools, which will further affect the formulation of treatment strategies [23]. The international guidelines recommend that clinicians can determine the extent of lymph node dissection based on FIGO staging, but the aggressive characteristic of NECC makes for a significantly higher incidence of LNM compared to cervical squamous cell carcinoma, and the overall survival rate of patients with LNM obviously decreases. Crucially, conventional models predominantly rely on anatomical parameters (such as tumor size, depth of stromal invasion) and ignore the potential predictive value of neuroendocrine markers. This limitation directly leads to two extremes in clinical practice: over‐treatment in low‐risk patients (such as extensive lymph node dissection) or under‐treatment in high‐risk patients [2]. Among other types of neuroendocrine tumors, there were researchers using NOVARA predictive score to assess nodal involvement risk of rectal neuroendocrine tumors to guide decision‐making in clinical practice [24]. And another research on appendiceal neuroendocrine neoplasms indicates accurate risk stratification is critical for optimizing surgical management and surveillance strategies [25]. As for NECC, the development of a prediction tool integrating multidimensional indicators is not only to address a lack of knowledge in precision medicine, but also to meet the urgent clinical need for optimizing individual therapeutic strategies, such as appropriate lymph node dissection and selection of neoadjuvant therapy.

In this present study, the results of univariate and multivariate logistic regression analyses showed that the deeper the stromal invasion, positive C‐UJI, positive LVSI and positive parametrium invasion, the higher the risk of LNM. In addition, based on the simplicity, rapidity and low cost of immunohistochemistry, immunohistochemical markers remain an important component of histopathological assessment. Therefore, this study also included three commonly used neuroendocrine markers which could be detected by immunohistochemistry, including Syn, CgA and CD56. Analysis revealed that patients with positive CD56 had an increased risk of LNM, so it was further incorporated into the present predictive model. CD56, known as neural cell adhesion molecule, is widely expressed in neuroendocrine neoplasms and plays a crucial role in regulating intercellular adhesion, signal transduction and cell motility [26]. Emerging evidence suggests that aberrant expression of CD56 may influence tumor aggressiveness by modulating intercellular adhesion and inducing the transformation of epithelial cells to the mesenchymal phenotype [27, 28]. In neuroendocrine tumors, over‐expression of CD56 has been associated with rapid progression and early metastasis by facilitating cellular motility and lymphovascular invasion [29, 30]. These biological features may explain the observed association between positive CD56 and LNM in NECC, supporting its value as a predictive marker in this present model.

The developed nomogram model enabled a specific prediction of the risk probability of LNM for each patient with NECC. For example, a patient with ≥ 1/2 cervical stromal invasion (67.5 points), positive C‐UJI (50 points), positive LVSI (98 points), positive parametrium invasion (57.5 points) and CD56 2+ (62.5 points) obtained a total score of 335.5 points, which corresponding probability of LNM was 0.85. This patient's probability of LNM was much > 0.20 (the optimal threshold of this model in risk stratification of LNM). The result suggested that clinicians should careful ly consider the necessity of performing systematic lymph node dissection for this patients. It showed that this developed model offered a “quantitatively” method to explain the risk level of LNM through a specific value, rather than just giving a simple conclusion that the risk of LNM was “high” or “low”. Meanwhile, after internal and external validation and comparison with other models, the C‐index of this developed model reached 0.894 and 0.920 in the training cohort and validation cohort respectively. It was significantly superior to the C‐index of the other models only relying on clinicopathological factors [20, 31]. It implied that the combination of specific markers with prognostic value and classical clinicopathological markers, such as immunohistochemical markers, could improve the prediction performance of the model.

The decision to perform lymphadenectomy depends on the primary lesion and the tumor characteristics of the lymph nodes. Theoretically, not only will patients without LNM not benefit from lymph node dissection, they will also suffer substantial surgical complications. Given the potentially higher frequency of adverse complications of lymph node dissection, we contend that the decision to perform lymphadenectomy should be predicated on an accurate and individualized assessment of the risk of LNM. The model established in this study determined the optimal threshold through the ROC curve to be 0.20, demonstrating a certain ability in differentiating the low‐risk patients (NPV = 95.2% in the validation cohort). This implies that the majority of low‐risk patients may avoid unnecessary lymph node dissection, thereby reducing surgical complications and enhancing quality of life. This finding contrasted with the study of Chen et al. [32], which performed risk stratification only based on surgical‐pathologic features and consequently failed to identify high‐risk patients with positive CD56. Therefore, this developed model to some extent may contribute to improving the ability to assess the risk probability of LNM in individual patients, so that individualized treatment strategies can be formulated to improve the overall prognosis of patients. Specifically, the model can assist clinicians in identifying patients with low‐risk LNM whose risk probability is < 0.20. These patients may be unlikely to undergo lymph node metastasis and therefore are recommended to avoid extensive lymphadenectomy as much as possible. Conversely, patients with a risk probability of higher than 0.20 are identified as high‐risk LNM by the model; they may warrant more comprehensive lymph node assessment, such as sentinel lymph node mapping or extended lymphadenectomy, as well as closer postoperative surveillance or consideration of intensified adjuvant therapy.

Of course, this developed model has some limitations. Firstly, given the rarity of NECC, the sample size of this study was small, which might lead to statistical bias to some extent. Although external validation (*n* = 92) partially addressed this issue, the rarity of NECC still restricted the establishment of a large sample cohort. Secondly, due to the high cost and inconvenience of molecular technology, the model did not incorporate molecular markers, such as TP53 or RB1, which had been proven to be associated with chemotherapy sensitivity in small cell carcinoma [33]. Then, another limitation is the lack of subgroup analyses for NECC, such as different stages or histological grades, due to the limited sample size and the low incidence of certain subgroups.

To address the above shortcomings, future research should focus on the following contents: Firstly, carry out multi‐center prospective research, particularly pay attention to the expression of CD56 in different patients and its impact on model calibration. Secondly, expand the screening of target proteins and search for new immunohistochemical markers or molecular markers. Research has highlighted the clinical applicability of these molecular markers, demonstrating their role in enhancing diagnostic accuracy, predicting surgical outcomes, and monitoring treatment responses [34]. Integrating genomic markers can enhance the biological interpretability of the nomogram model and may improve its predictive accuracy, especially in stratifying patients according to treatment responsiveness or metastatic potential. As generation sequencing becomes increasingly accessible in clinical practice, future studies should explore whether adding these molecular features strengthens the model's discriminatory ability and calibration, and whether a combined clinico‐molecular prediction model will provide more robust guidance for individualized risk assessment and management in NECC. Thirdly, investigations involving larger, multi‐center cohorts are warranted in the future to enable reliable subgroup analyses and validate the practicality of the model across diverse clinical classification, and further explore potential interactions between tumor biology, disease stage, and metastatic propensity.

In conclusion, the nomogram model constructed in this study preliminarily provides a potential tool for the assessment of LNM risk in NECC. Its application in clinical practice may contribute to developing a more personalized treatment. Of course, this model still requires further refinement. With the development of artificial intelligence, it is possible to integrate more factors such as radiomics and genomics in the future to assist the formulation of individualized management for patients with NECC.

## Author Contributions

**Mingzhu Jia:** conceptualization, data curation, investigation, formal analysis, writing – original draft. **Siyuan Zeng:** data curation, investigation, formal analysis. **Juan Zou:** data curation, investigation. **Huiling Chen:** data curation, investigation. **Changsheng Lin:** data curation, investigation. **Shuqi Yang:** data curation, investigation. **Jiangchuan Pi:** conceptualization, methodology, writing – review and editing. **Xue Xiao:** conceptualization, writing – review and editing.

## Ethics Statement

The research was conducted in accordance with the ethical principles of the Declaration of Helsinki and received approval from the ethics committees of West China Second Hospital of Sichuan University and the First Affiliated Hospital of Chongqing Medical University.

## Consent

Informed consent was obtained from all participants prior to their inclusion in this study.

## Conflicts of Interest

The authors declare no conflicts of interest.

## Data Availability

The data that support the findings of this study are available on request from the corresponding author. The data are not publicly available due to privacy or ethical restrictions.
